# Mechanisms mediating muscle metaboreflex control of cardiac output during exercise: Impaired regulation in heart failure

**DOI:** 10.1113/EP091752

**Published:** 2024-03-09

**Authors:** Donal S. O'Leary, Joseph Mannozzi

**Affiliations:** ^1^ Department of Physiology Wayne State University School of Medicine Detroit Michigan USA

**Keywords:** baroreflex, exercise pressor reflex, systolic failure, ventricular contractility, ventricular function

## Abstract

The ability to increase cardiac output during dynamic exercise is paramount for the ability to maintain workload performance. Reflex control of the cardiovascular system during exercise is complex and multifaceted involving multiple feedforward and feedback systems. One major reflex thought to mediate the autonomic adjustments to exercise is termed the muscle metaboreflex and is activated via afferent neurons within active skeletal muscle which respond to the accumulation of interstitial metabolites during exercise when blood flow and O_2_ delivery are insufficient to meet metabolic demands. This is one of the most powerful cardiovascular reflexes capable of eliciting profound increases in sympathetic nerve activity, arterial blood pressure, central blood volume mobilization, heart rate and cardiac output. This review summarizes the mechanisms meditating muscle metaboreflex‐induced increases in cardiac output. Although much has been learned from studies using anaesthetized and/or decerebrate animals, we focus on studies in conscious animals and humans performing volitional exercise. We discuss the separate and interrelated roles of heart rate, ventricular contractility, ventricular preload and ventricular–vascular coupling as well as the interaction with other cardiovascular reflexes which modify muscle metaboreflex control of cardiac output. We discuss how these mechanisms may be altered in subjects with heart failure with reduced ejection fraction and offer suggestions for future studies.

## Abstract (highlights)

- **What is the topic of this review?**
  This review summarizes the mechanisms meditating muscle metaboreflex‐induced increases in cardiac output—one of the most powerful cardiovascular reflexes capable of eliciting marked increases in cardiac output, which raises arterial blood pressure and increases blood flow to the ischaemic muscles.
- **What advances does it highlight?**
  The ability to raise cardiac output is severely impaired in heart failure: this review discusses the interrelated roles of heart rate, ventricular contractility, ventricular preload and ventricular–vascular coupling, the interactions with other cardiovascular reflexes, and how these mechanisms may be altered in subjects with heart failure with reduced ejection fraction.

## 1 INTRODUCTION

Whole body dynamic exercise presents one of the greatest challenges to cardiovascular control. If exercise is to be sustained, cardiac output must increase to provide blood flow and O_2_ delivery to the active skeletal muscles as well as protect arterial blood pressure in the face of a profound increase in total vascular conductance due to the massive skeletal muscle vasodilatation. One of the primary mechanisms mediating sustained increases in cardiac output is via a reflex increase in sympathetic activity elicited by the activation of metabolite‐sensitive afferents within the active skeletal muscles—termed the muscle metaboreflex. This reflex is among the strongest cardiovascular reflexes, rivalling the pressor responses to cerebral ischaemia in the ability to increase arterial blood pressure (Sheriff et al., 1990). This is a flow‐sensitive–flow‐raising reflex. It is activated in response to a mismatch between O_2_ delivery and O_2_ demand (Sheriff et al., 1987), and one of the primary responses is to raise total systemic blood flow, that is, cardiac output, which partially restores blood flow (O'Leary & Sheriff, 1995) and O_2_ delivery (O'Leary et al., 1999) to the under‐perfused active muscle. In subjects with heart failure with reduced ejection fraction (HFrEF, or more simply for this review abbreviated HF), the muscle metaboreflex is markedly altered and the ability to raise cardiac output is severely attenuated (Coutsos et al., 2013; Crisafulli et al., 2007; Hammond et al., 2000; Notarius et al., 2001). This review is focused on the mechanisms mediating muscle metaboreflex control of cardiac output and how these are altered in HF. Much of what we have learned regarding the muscle metaboreflex, especially in terms of afferent sensitivities and central processing, has been learned using anaesthetized and/or decerebrate animal preparations. However, to our knowledge, no study using these preparations has ever measured cardiac output. Given that the vast majority of these studies used strong static muscle contractions or infusion of drugs into the muscles to activate the metaboreflex afferents, and that the heart rate responses are often modest at best, it is unlikely substantial sustained increases in cardiac output occurred. However, this has yet to be investigated. This review is mainly limited to observations in humans and/or conscious, chronically instrumented canines during volitional exercise.

## 2 EFFERENT MECHANISMS MEDIATING THE MUSCLE METABOREFLEX

**FIGURE 1:**
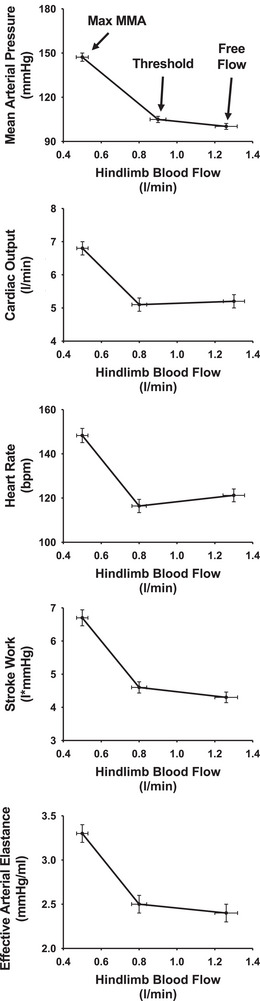
Average metaboreflex responses during mild exercise (3.2 km/h 0% grade) from 36 animals. Data points are during free flow exercise (no occlusion; Free Flow), the metaboreflex threshold (Threshold) determined as the intersection between the initial response line and the pressor response line, and the maximal value observed with the lowest level of occlusion performed during that experiment (Max MMA).

Many if not most studies investigating the muscle metaboreflex in humans have used the technique of post‐exercise muscle ischaemia (PEMI). In this setting, the subject performs exercise with an arm or leg (usually static or intermittent static contractions), and then at the cessation of exercise, blood flow to the limb is rapidly occluded thereby entrapping metabolites within the formally active skeletal muscle, which sustains the activation of metaboreceptor afferents. While a large amount of information has been gleaned using this technique (including the actual discovery of this reflex by Alam & Smirk, 1937), one major limitation is that in the cardiovascular responses are observed during the recovery from exercise rather than during the exercise itself (O'Leary, 1993). This is important because during PEMI, whereas arterial pressure remains elevated due to the sustained muscle metaboreflex activation (MMA), heart rate decreases with a time course similar to the normal recovery from exercise, which led to the idea that the muscle metaboreflex does not have strong control over heart rate (O'Leary, 1996). However, in subsequent studies using chronically instrumented canines trained to run on a treadmill, the muscle metaboreflex was activated during dynamic exercise via imposed reductions in leg blood flow, and substantial increases in arterial pressure and heart rate occurred (Wyss et al., 1983). Indeed, the primary mechanism mediating the rise in mean arterial pressure is the reflex increase in cardiac output primarily driven by robust increases in heart rate with sustained or slightly increased stroke volume (Hammond et al., 2000; Mannozzi et al., 2023; Sheriff et al., 1998; Wyss et al., 1983). Figure 1 shows the average results from experiments performed in our laboratory over the last ∼15 years using 36 chronically instrumented canines. Animals were instrumented to allow graded acute reductions in blood flow to the hindlimbs during exercise. During mild exercise (3.2 km/h 0% grade), blood flow must be reduced below a critical threshold level before the muscle metaboreflex is activated. Thereafter, linear increases in mean arterial pressure, heart rate, cardiac output, stroke work and arterial elastance occur. As workload increases, this threshold moves closer and closer to the normal prevailing level of hindlimb blood flow, and at moderate workloads often no threshold is observed indicating that this reflex is tonically active (Augustyniak et al., 2001; Hammond et al., 2000; Wyss et al., 1983). The strength or gain of the muscle metaboreflex has been quantified by the slope of the relationships between the change in skeletal muscle blood flow and the subsequent reflex changes in the various cardiovascular parameters. Interestingly, the slope of the relationship between hindlimb blood flow and cardiac output presents a true unitless gain in classic control systems theory as both are expressed in the same units of flow (Sagawa, 1983). In Figure 1, this slope averaged 7.7. This means that for every 100 ml/min reduction in hindlimb blood flow beyond metaboreflex threshold, cardiac output increased 770 ml/min. An open loop gain of 7.7 is exceedingly high for biological control systems. In comparison, the unitless open loop gain of the arterial baroreflex is usually ∼ 1.0–2.0 (assessed using an isolated carotid sinus model in which the changes in systemic arterial blood pressure are observed in response to changes in pressure at the isolated carotid sinus baroreceptors (Stephenson & Donald, 1980a, b). To our knowledge, this is the highest gain ever reported for control of cardiac output by a biological reflex control system. This rivals gain values for arterial pressure in response to cerebral ischaemia seen in classic studies from Guyton's laboratory (Sagawa et al., 1961), which is generally regarded as the strongest cardiovascular reflex (average gain = 7.7).

The disparity between the heart rate observations during PEMI versus those with MMA performed during dynamic exercise likely stems from the different baseline levels of autonomic activity in each setting as well as the autonomic mechanisms mediating the changes in heart rate (O'Leary, 1993, 1996). When the muscle metaboreflex is activated during dynamic exercise, the primary mechanism mediating the rise in heart rate is via activation of the sympathetic nerves to the heart (O'Leary, 1993). During PEMI, parasympathetic tone rises with the recovery from exercise, which overwhelms the effect of sustained sympathetic activation, and heart rate declines in both humans and canines (Fisher et al., 2010; Nishiyasu et al., 1994; O'Leary, 1993; O'Leary, 1996). If PEMI is performed after parasympathetic blockade in canines, the increased heart rate is sustained for as long as the occlusion of flow to muscle is maintained (O'Leary, 1993). Finally, in addition to reflex changes in autonomic activity, increases in circulating levels of the vasoactive hormones vasopressin and angiotensin II may also contribute to the pressor response to muscle metaboreflex activation (Nishiyasu et al., 1998; O'Leary et al., 1993).

## 3 MECHANISMS MEDIATING METABOREFLEX‐INDUCED INCREASES IN CARDIAC OUTPUT

Factors affecting cardiac output control include heart rate, stroke volume, ventricular contractility, preload and afterload. These are often co‐dependent as each factor may affect another. As discussed above, MMA increases sympathetic activity to the heart (O'Leary, 1993). If unopposed by substantial parasympathetic tone, this causes increases in heart rate as is seen with MMA during dynamic exercise or PEMI performed after parasympathetic blockade (Augustyniak et al., 2001; O'Leary, 1993; O'Leary & Augustyniak, 1998; O'Leary & Seamans, 1993). However, we and others have shown that there are sustained increases in cardiac output during PEMI in both humans and canines (Crisafulli et al., 2003; Rowell et al., 1981; Shoemaker et al., 2007; Spranger et al., 2013). Furthermore, the increases in cardiac output during MMA may not even require an increase heart rate. When heart rate is maintained constant with MMA during dynamic exercise via pacing the heart at levels well above any reflex tachycardia, similar increases in cardiac output occurred (O'Leary & Augustyniak, 1998). Normally, with MMA during dynamic exercise the substantial increases in cardiac output are achieved via tachycardia with sustained or slightly increased stroke volume (Augustyniak et al., 2001; Crisafulli et al., 2011; Hammond et al., 2000; Mannozzi et al., 2023; O'Leary & Augustyniak, 1998; Rowell et al., 1991). With MMA during constant heart rate, the same increase in cardiac output occurred but now this increased cardiac output occurred via large increases in stroke volume with sustained heart rate (O'Leary & Augustyniak, 1998). Inasmuch as filling pressure remained unchanged, these results strongly indicated that MMA caused substantial increases in ventricular contractility which raised cardiac output at constant heart rate. Subsequently, we showed that MMA during exercise in canines causes marked increases in left ventricular maximal elastance (*E*_max_) as well as preload recruitable stroke work, which are two superb indices of ventricular contractility (Sala‐Mercado, Hammond, Kim, Rossi et al., 2006). Similar increases in left ventricular maximal and minimal d*P*/d*t* as well as myocardial segment shortening speed occurred (Ansorge et al., 2005; Sala‐Mercado, Hammond, Kim, McDonald et al., 2006; Sala‐Mercado, Hammond, Kim, Rossi et al., 2006).

Increases in heart rate and ventricular contractility likely cannot sustain increases in cardiac output alone, inasmuch as with increases in cardiac output, there is translocation of blood volume from the venous to the arterial circulation and ventricular preload falls. This inverse relationship between cardiac output and ventricular preload observed in many anaesthetized animal studies using cardiac bypass was recapitulated by Sheriff et al. (1993) in conscious canines at rest and during exercise. Thus, to sustain large increases in cardiac output, substantial central blood volume mobilization must occur. We utilized constant ventricular rate coupled with β‐adrenergic blockade (to limit increases in inotropicity), which combined prevented metaboreflex‐induced increases in cardiac output, and in this setting during MMA substantial increases in right atrial pressure occurred (Sheriff et al., 1998). This central blood volume mobilization seen with MMA was over three times greater than that observed with baroreflex activation via bilateral carotid occlusion (Bennett et al., 1984). Thus, not only is the muscle metaboreflex one of the strongest reflexes in the ability to raise cardiac output and arterial pressure, it is also one of the strongest in the ability to raise central venous pressure. This ability to sustain left ventricular preload during MMA is likely a key factor allowing the increased ventricular contractility to maintain or increase stroke volume, which in combination with the reflex tachycardia causes the large increases in cardiac output. To what extent the large metaboreflex increases in mean arterial pressure suppress the rise in cardiac output due to the increased ventricular afterload is unclear. Even with substantial increases in ventricular afterload during MMA, stroke volume is well maintained or even increased slightly, likely due to the marked increase in ventricular contractility. The role of afterload in limiting MMA‐induced increases in cardiac output has yet to be investigated.

## 4 ALTERED MUSCLE METABOREFLEX CONTROL OF CARDIAC OUTPUT IN SYSTOLIC HEART FAILURE

**FIGURE 2:**
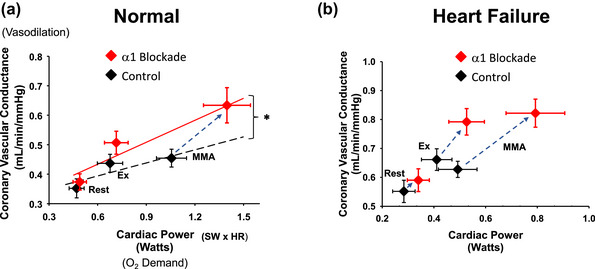
(a) The relationship between coronary vascular conductance (CVC) and cardiac power at rest, during mild exercise (Ex) and during muscle metaboreflex activation (MMA) before and after i.v. infusion of the α1‐adrenergic antagonist prazosin. In control experiments, coronary vasodilatation occurred in response to exercise and an increase in cardiac power. In response to MMA, cardiac power increased with little change in CVC. Prazosin had little effect at rest or during exercise, but with MMA marked increases in CVC occurred in response to much greater increases in cardiac power. The slope of this relationship was significantly increased after prazosin. (b) This same experiment after induction of HF. See text for explanations. Adapted from Coutsos et al. 2010, 2013).

**FIGURE 3:**
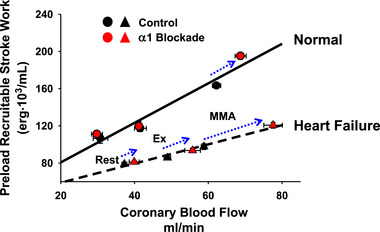
The relationship between an index of cardiac contractility (preload recruitable stroke work) versus coronary blood flow at rest, during mild exercise and during muscle metaboreflex activation before and after prazosin and in the same animals before and after the induction of heart failure (abbreviations as in Figure 2). In normal animals prazosin extends the linear relationship to higher levels of coronary blood flow and higher cardiac contractility (dashed blue arrow). After induction of HF, the slope of the relationship is significantly lower. Prazosin had a progressively greater effect as baseline coronary blood flow increased. With MMA, prazosin had a marked effect on flow and contractility increased significantly. Adapted from Coutsos et al. (2010, 2013).

A hallmark of HF is reduced exercise tolerance. There is depressed cardiac output at rest, and increases during exercise are limited. The most obvious explanation for the reduced ability to raise cardiac output during exercise in HF is that the inherent ventricular dysfunction reduces the ability of the heart to generate tension as well as respond to sympathetic activation. However, even in normal individuals during exercise the rise in sympathetic activity to the heart activates both myocardial β‐adrenergic receptors and coronary vascular α‐adrenergic receptors (Gwirtz et al., 1986; Huang & Feigl, 1988). Stimulation of these α‐receptors activates coronary arterial vascular smooth muscle, which thereby limits coronary vasodilatation during exercise. In an elegant study, Gwirtz and colleagues infused the α‐adrenergic antagonist prazosin into the circumflex coronary artery in conscious canines trained to run on a treadmill (Gwirtz et al., 1986). This caused greater myocardial vasodilatation during exercise, and the left ventricular myocardium perfused by this artery showed increased ventricular inotropicity. This indicates that the normal increase in sympathetic activity to the heart during exercise functionally restrains coronary blood flow and O_2_ delivery to the myocardium, which thereby limits the ability to raise ventricular function. We showed that in the normal canine, i.v. prazosin raises coronary vascular conductance and ventricular contractility during exercise and MMA and that larger metaboreflex‐induced increases in cardiac output occur (Coutsos et al., 2010, 2013). To address the relationships between coronary vasodilatation and cardiac work versus cardiac work and coronary blood flow, we analysed our data similarly to Feigl (1983) by plotting coronary vascular conductance (vasodilatation) as a function of cardiac power (an index of myocardial O_2_ consumption) (Figure 2). Figure 2a shows that prazosin had little effect on coronary vasodilatation at rest and led to a small vasodilatation with little change in cardiac power during mild exercise, but with MMA much greater increases in both coronary vasodilatation and myocardial power occurred (dashed blue arrow) and the slope of this relationship was much greater (Coutsos et al., 2010). This greater slope indicates that the increased coronary vasodilatation with prazosin was not simply due to the rise in cardiac work, as vasodilatation would have only increased modestly if cardiac work had increased to the same levels as in the control experiments (control regression line). Figure 2b shows this same relationship after the induction of HF. MMA during mild exercise in HF causes frank coronary vasoconstriction as a significant decrease in coronary vascular conductance occurs (Ansorge et al., 2005; Coutsos et al., 2013). This attenuation of increases in coronary blood flow greatly limits the ability to raise cardiac power (Coutsos et al., 2013). In this setting, prazosin causes significant increases in coronary vascular conductance even during mild exercise and substantial further increases occurred with MMA, which allows for much greater increases in O_2_ delivery and cardiac power (dashed blue arrows), and the ability to raise cardiac output is significantly improved (Coutsos et al., 2013). Figure 3 shows the relationship between an index of cardiac contractility (preload recruitable stroke work) versus coronary blood flow at rest, during mild exercise, and during MMA before and after prazosin and in the same animals before and after the induction of heart failure (Coutsos et al., 2013). All of these individual points within a given condition (Normal vs. HF) lie on the same linear relationship. In normal animals, prazosin had little effect at rest or during mild exercise; however, during MMA, larger increases in coronary blood flow occurred, which extended this relationship to higher levels of contractility. Comparison of the contractility–coronary blood flow relationships in normal and HF shows that the slope of this relationship is substantially reduced in HF. However, the effects of prazosin are much greater in HF, and during MMA prazosin extend the relationship much more to higher levels of coronary blood flow and cardiac contractility. Indeed, with prazosin the ability to raise cardiac output during MMA is partially restored (Coutsos et al., 2013). These data strongly indicate that the depressed ability to raise ventricular contractility and cardiac output during MMA in HF stems not only from the inherent cardiac dysfunction, but also from increased coronary vasoconstriction which restricts O_2_ delivery to the myocardium and impairs the ability to raise inotropic state. These data strongly indicate that if α‐adrenergic receptor antagonists could be chronically administered to only the coronary vasculature, the ability to raise coronary blood flow with even mild exercise would be improved. This potential treatment could be investigated.

In HF there is increased activation of the cardiac sympathetic afferent reflex, which is elicited by stimulation of sympathetic afferent neurons in the myocardium and causes increases in efferent sympathetic activity as well as depressed arterial baroreflex activation (W. Wang & Zucker, 1996; W. Wang et al., 1999; H. J. Wang et al., 2014). Inasmuch as we have shown that there is excessive activation of the sympathetic activity to the heart during MMA in HF and that this increased sympathetic tone causes exaggerated coronary vasoconstriction during exercise (Coutsos et al., 2013), we hypothesized that during MMA the resultant coronary vasoconstriction could further stimulate the cardiac sympathetic afferent reflex, which would cause even further sympatho‐activation. This would effectively serve as an amplifier of the responses to MMA. Using the techniques described by H. J. Wang et al. (2014), we chronically denervated the ventricles of cardiac sympathetic afferents by painting the tissue with resiniferatoxin (RTX), an ultra‐potent agonist of TRPV1 receptors, which are present on these sympathetic afferents (H. J. Wang et al., 2014). RTX will irreversibly bind to TRPV1 receptors causing Ca^2+^ influx, which destroys the afferents. We found that after destruction of the cardiac sympathetic afferents, arterial blood pressure and cardiac output responses to MMA were reduced both before and after the induction of HF indicating that MMA likely activates the cardiac sympathetic afferent reflexes, which amplifies the metaboreflex responses (Mannozzi et al., 2023). Thus, the heart is both a target as well as a source of sympathetic efferent activity.

**FIGURE 4:**
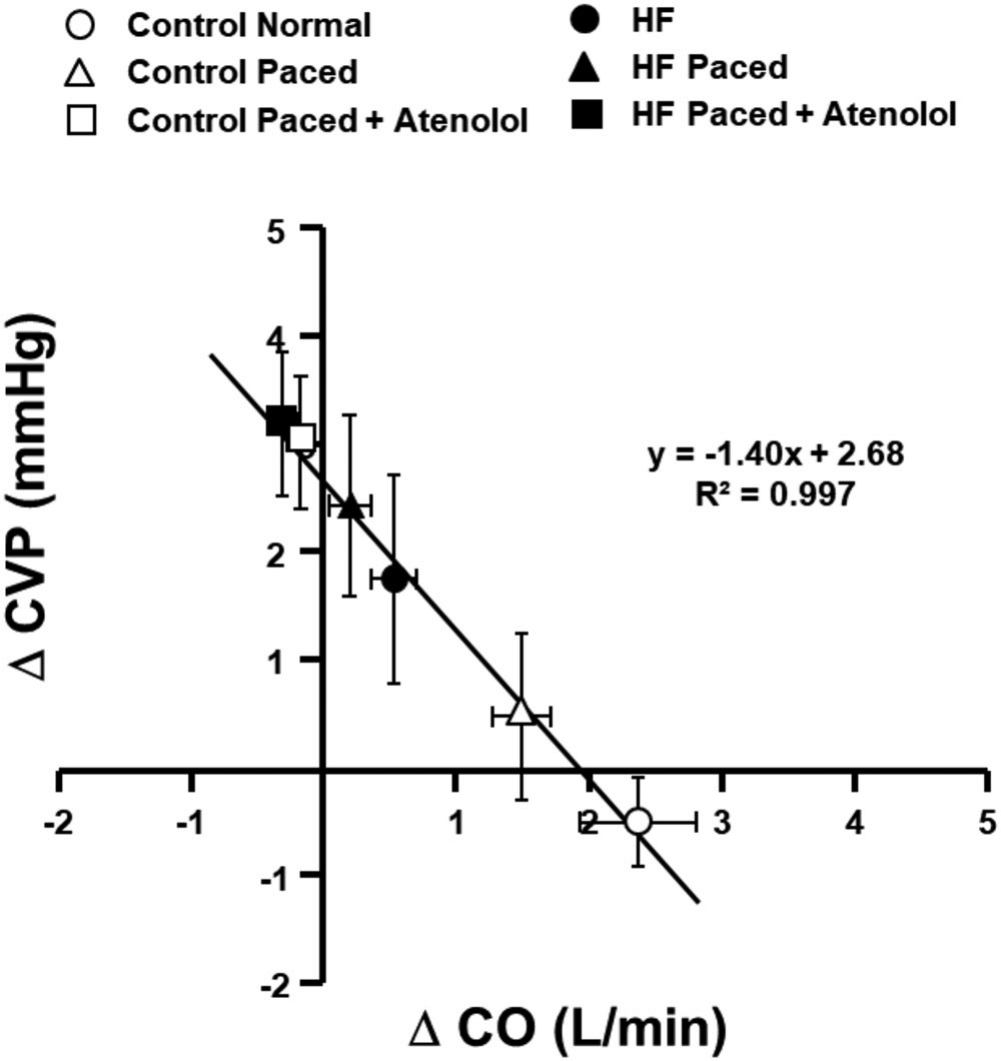
The relationship between the changes in central venous pressure (CVP) in response to MMA as a function of the observed change in cardiac output before and after the induction of HF. As the rise in cardiac output is attenuated, larger increases in CVP occur during MMA. Adapted from O'Leary et al. (2019).

Since sustained increases in cardiac output can only occur if ventricular preload is maintained (Sheriff et al., 1993; Sheriff et al., 1998), we investigated whether inability to raise central blood volume mobilization plays any role in limiting the ability to raise cardiac output during MMA in HF. We activated the muscle metaboreflex in canines before and after the induction of HF in normal conditions, with ventricular rate held constant and with constant heart rate coupled with β‐adrenergic blockade (O'Leary et al., 2019). Figure 4 shows the relationship between the changes in central venous pressure (CVP) with MMA as a function of the observed change in cardiac output. During control experiments prior to the induction of HF, MMA caused large increases in cardiac output, and only a small, non‐significant decreases in CVP occurred. Inasmuch as with this large increase in cardiac output preload to the heart should have decreased (Sheriff et al., 1993), the observation that large increases in cardiac output occur with little change in CVP is indirect evidence that MMA increases central blood volume mobilization. With constant pacing in normal animals, similar increases in cardiac output occurred with little change in CVP. When β‐adrenergic blockade using atenolol was added to the constant pacing in normal animals, the rise in cardiac output was abolished and a large increase in preload occurred, which directly shows substantial central blood volume mobilization. During control experiments after the induction of HF, the rise in cardiac output was much smaller and a significant increase in CVP occurred during MMA. With constant pacing, a trivial increase in cardiac output occurred during MMA and the rise in CVP was even greater. Finally, with β‐adrenergic blockade in HF, MMA caused a very small decrease in cardiac output and CVP increased even further. Note that all of these points lie on the same linear regression line showing that the relationship between the changes in CVP and the changes in cardiac output are similar. These data indicate that the inability to raise cardiac output in HF during MMA likely does not stem from the inability to sustain preload.

Whereas overall ventricular preload is sustained similarly in HF as in normal subjects, we investigated whether impaired stroke volume and cardiac output during MMA in HF may be also due to altered contraction/relaxation dynamics (Mannozzi et al., 2021), similar to what has been shown in studies using single sarcomeres (Janseen, 2010a, 2010b). We observed that in normal subjects during exercise, ventricular dynamics favour increased contraction rate as opposed to relaxation rate. With MMA there is a robust shift in this relationship drastically favouring the rate of relaxation relative to contraction. This suggests that sympathetic activation to the heart not only improves contractile function but also significantly increases ventricular relaxation, which would aid ventricular filling. In heart failure, this relationship is stagnant, and the robust improvements in ventricular relaxation relative to contraction observed in normal animals are completely abolished. This likely contributes to the impaired ability to raise cardiac output during exercise in HF.

## 5 VENTRICULAR–VASCULAR COUPLING

One of the greatest challenges to improving cardiac performance during exercise is the maintenance of optimal energy transfer from the left ventricle to the systemic circulation, termed ventricular–vascular coupling. Initial studies by Sunagawa et al. (1983, 1985) pioneered our understanding of this delicate interaction and provided a framework for evaluating ventricular–vascular coupling. Since then, the metrics that evaluate ventricular–vascular coupling have been simplified to the relationship between effective arterial elastance (*E*_a_), an index of arterial load, and left ventricular maximal elastance (*E*_max_), an index of ventricular contractility. More simply put, the ventricular component, *E*_max_, is an assessment of energy generation and its subsequent transfer to the systemic circulation, whereas the arterial component, *E*_a_, is an assessment of accommodation and propagation of that energy transfer throughout the arterial tree. The combination of these metrics together allows for the evaluation of ventricular–vascular coupling through examination of the ventricular–vascular coupling ratio (VVCR) determined as *E*_a_/*E*_max_, which has been shown to be most optimal in a range of 0.6–1.2 (Chantler et al., 2008). Outside of this range, ventricular–vascular uncoupling occurs indicating impaired energy transfer from the left ventricle to the systemic circulation (Chantler et al., 2008).

With MMA eliciting alterations in both ventricular and vascular properties during exercise, it begs the question if activation of this reflex acted to improve coupling to maintain workload performance or hindered it such that it might induce fatigue and limitations on the duration of workload performance. Utilizing data across two different studies as well as an index of ventricular–vascular coupling efficiency, stroke work (Chantler et al., 2008; Najjar et al., 2004), we observed that MMA induces significant increases in *E*_a_ that are well matched by robust improvements in *E*_max_ leading to robust increases in stroke work (Mannozzi et al., 2020; Sala‐Mercado, Hammond, Kim, McDonald et al., 2006). Thus, during MMA, energy transfer from the left ventricle to the systemic circulation is sustained at optimal levels, which thereby ensures effective propagation of cardiac output throughout the arterial system, which maintains or even improves perfusion pressure to exercising muscle.

In HF the ventricular–vascular relationship is already uncoupled at rest since ventricular contractility is reduced and arterial stiffness is increased (Ahmadian et al., 2022; Coutsos et al., 2013; Mannozzi et al., 2020; Mannozzi, Al‐Hassan et al., 2022; Sala‐Mercado, Hammond, Kim, McDonald et al., 2006). With exercise and MMA in HF, little increase in ventricular inotropic state occurs and the normal increases in *E*_a_ are substantially greater (Mannozzi et al., 2020; Mannozzi, Al‐Hassan et al., 2022; Sala‐Mercado, Hammond, Kim, McDonald et al., 2006). Together both effects lead to a profound reduction in the ability to improve stroke work, and thus MMA in heart failure leads to exaggeration of the ventricular–vascular uncoupling thereby worsening an already impaired skeletal muscle perfusion during exercise (Mannozzi, Al‐Hassan et al., 2022). However, MMA activation may not be the sole contributor to the exaggerated ventricular–vascular uncoupling. The arterial baroreflex is known to buffer muscle metaboreflex‐induced peripheral vasoconstriction (Kim, Sala‐Mercado, Rodriguez et al., 2005). During MMA after sino‐aortic baroreceptor denervation (SAD) in a normal canine, *E*_a_ is increased significantly relative to baro‐intact animals; however, the ability to improve stroke work is maintained (Mannozzi, Kim et al., 2022). This suggests that with healthy ventricular function, *E*_max_ can rise to meet the increases in *E*_a_ to maintain normal ventricular–vascular coupling. However, in heart failure after SAD, an even greater increase in *E*_a_ occurs versus those observed in healthy subjects and is accompanied by a significantly impaired ability to raise stroke work (Mannozzi, Kim et al., 2022). This suggests that baroreflex buffering capacity may play a role in determining the degree of ventricular–vascular uncoupling during MMA. This may be crucial since many studies have suggested that arterial baroreflex strength is reduced in HF (Iellamo et al., 2007; Kim et al., 2004; Kim, Sala‐Mercado, Hammond et al., 2005; Marin‐Neto et al., 1991; Mortara et al., 1997; Sala‐Mercado et al., 2008; Zucker & Wang, 1991).

## 6 EFFECT OF HEART FAILURE ON METABOREFLEX STRENGTH

To what extent the strength (gain) of the muscle metaboreflex is impaired in HF is somewhat dependent on the method of analysis of the metaboreflex responses. When activated during dynamic exercise in normal subjects, the major mechanism mediating the metaboreflex pressor response is the large rise in cardiac output (Augustyniak et al., 2001; Eiken & Bjurstedt, 1987; Hammond et al., 2000; Kim, Sala‐Mercado, Rodriguez et al., 2005; Rowell et al., 1991). After the induction of HF, the ability to raise cardiac output is markedly attenuated and the reflex shifts to substantial peripheral vasoconstriction (Hammond et al., 2000; Kaur, Senador et al., 2018; Kim, Sala‐Mercado, Hammond et al., 2005). Thus, when analysed in terms of the ability to raise cardiac output, the strength of the metaboreflex is markedly attenuated in HF. However, when analysed in terms of the ability to induce peripheral vasoconstriction, the gain of the metaboreflex is enhanced. When analysed in terms of the ability to raise arterial blood pressure, the reflex gain is generally maintained or slightly reduced in HF (Augustyniak et al., 2006; Kim, Sala‐Mercado, Hammond et al., 2005; Mannozzi et al., 2023). Metaboreflex activation after induction of HF in canines results in substantially larger increases in the arterial plasma levels of noradrenaline indirectly indicating larger increases in sympathetic nerve activity (Hammond et al., 2000). Why the mechanisms of the reflex shift from raising cardiac output to eliciting vasoconstriction is unclear but may be due to attenuated buffering by the arterial baroreflex in HF (Kim, Sala‐Mercado, Hammond et al., 2005). In normal subjects, SAD causes a marked increase in the strength of the metaboreflex in the ability to raise arterial blood pressure (Kim, Sala‐Mercado, Rodriguez et al., 2005; Sheriff et al., 1990). This is due to substantial peripheral vasoconstriction now accompanying increases in cardiac output (Kim, Sala‐Mercado, Rodriguez et al., 2005). In HF, baroreflex function is attenuated, and SAD does not markedly affect the pattern of the metaboreflex (Kim, Sala‐Mercado, Hammond et al., 2005). The reflex has already shifted to peripheral vasoconstriction in HF, and after SAD this vasoconstriction is somewhat increased. Thus, it appears that in normal subjects, the arterial baroreflex mainly buffers the muscle metaboreflex via inhibiting muscle metaboreflex‐induced peripheral vasoconstriction.

With depressed baroreflex function in HF, the ability to buffer the metaboreflex is attenuated and substantial peripheral vasoconstriction now occurs (Kim, Sala‐Mercado, Hammond et al., 2005). Since during dynamic exercise most of the cardiac output is directed to the active skeletal muscle, substantial functional peripheral vasoconstriction can only occur via constriction of the active skeletal muscle (Collins et al., 2001; Kaur et al., 2015; Kaur, Krishnan et al., 2018; Kaur, Senador et al., 2018; Kim et al., 2004; Kim, Sala‐Mercado, Hammond et al., 2005; Kim, Sala‐Mercado, Rodriguez et al., 2005; O'Leary, 1991). Marked vasoconstriction of inactive vascular beds such as the kidney will have progressively smaller and smaller effects on mean arterial pressure since, as workload rises, vascular conductance to inactive beds constitutes a smaller and smaller fraction of total vascular conductance (Collins et al., 2001; O'Leary, 1991). Indeed, we have shown that even in normal animals during MMA there is some vasoconstriction of even the ischaemic muscle from which the reflex arises, and in HF this is markedly exaggerated (Kaur et al., 2015; Kaur, Senador et al., 2018). This creates a positive‐feedback amplification of the muscle metaboreflex responses and this amplification is increased during HF (Kaur et al., 2015; Kaur, Senador et al., 2018).

Skeletal muscle afferents are often polymodal and respond to physical changes as well as chemical substances (Hayes et al., 2005; Kaufman & Rybicki, 1987; Kaufman et al., 1983; Kaufman et al., 1988; Rotto & Kaufman, 1988). These afferents are richly endowed with a variety of receptors/channels which may be activated during exercise and MMA (Kaufman & Rybicki, 1987; Kaufman et al., 1988). The expression of several of these receptors is significantly altered in HF (Antunes‐Correa et al., 2014; S. A. Smith et al., 2005; J. R. Smith et al., 2020; H. J. Wang et al., 2010; Xing & Li, 2016; Xing et al., 2015). Thus, the number and composition of skeletal muscle afferents, production of metabolite by‐products during exercise as well as the extent of physical forces generated in the muscle during contractions may all change in HF in complex fashions. Finally, the central processing as well as the strength of the end‐organ responses to changes in autonomic and hormonal responses to MMA may vary in HF. These important considerations are beyond the scope of this review and deserve detailed future analyses of our current understanding.

## 7 CONCLUSIONS

We conclude that in normal subjects during submaximal exercise, the muscle metaboreflex is a flow sensitive–flow raising reflex. It is stimulated by under‐perfusion of the active skeletal muscle and responds by raising total systemic blood flow—cardiac output—which partially relieves the blood flow and O_2_ delivery debt to the under‐perfused active skeletal muscle (O'Leary & Sheriff, 1995; O'Leary et al., 1999). When the ability to raise cardiac output is limited as in HF or when workload approaches maximal, the muscle metaboreflex shifts to eliciting substantial peripheral vasoconstriction (Augustyniak et al., 2001; Crisafulli et al., 2007; Hammond et al., 2000; Ichinose et al., 2010; Kaur, Krishnan et al., 2018; Kaur, Senador et al., 2018; Kim, Sala‐Mercado, Hammond et al., 2005). Even the active skeletal muscle is relatively vasoconstricted (Kaur, Senador et al., 2018). In this setting, whether less vasoconstriction occurs in the most ischaemic muscle, which would thereby redistribute the limited cardiac output to the most ischaemic muscle, is unclear and requires further investigation (Rowell & Sheriff, 1988). The studies cited investigating MMA after induction of HF in canines all used the rapid ventricular pacing model of HFrEF. To what extent these results replicate those seen in humans with HFrEF due to other issues (e.g. coronary artery disease, other myopathies, etc.) is unclear. Studies by Crisafulli and colleagues in humans with HF observed very similar responses to those in the canine studies—in the normal condition the metaboreflex functions to raise cardiac output and in HF the reflex converts to peripheral vasoconstriction (Augustyniak et al., 2006; Crisafulli & Concu, 2007; Crisafulli et al., 2007; Hammond et al., 2000; Kim, Sala‐Mercado, Hammond et al., 2005). However, other animal models of HFrEF deserve further investigation.

## AUTHOR CONTRIBUTIONS

Donal S. O'Leary and Joseph Mannozzi drafted the manuscript. Donal S. O'Leary and Joseph Mannozzi contributed to the ideas within the manuscript. Both authors contributed to revisions of the manuscript. Both authors have read and approved the final version of this manuscript and agree to be accountable for all aspects of the work in ensuring that questions related to the accuracy or integrity of any part of the work are appropriately investigated and resolved. All persons designated as authors qualify for authorship, and all those who qualify for authorship are listed.

## CONFLICT OF INTEREST

The authors declare no conflicts of interest.
